# Inotuzumab Ozogamicin in Clinical Practice: an Overview of Efficacy, Safety, and Real-World Applications

**DOI:** 10.1007/s11899-026-00772-7

**Published:** 2026-02-24

**Authors:** Valerie Tran, Nandan Srinivasa, Caroline Tatum, Daniel Reed, Michael Keng

**Affiliations:** 1 Department of Medicine, University of Virginia, Charlottesville, VA USA; 2 Department of Pharmacy, University of Virginia, Charlottesville, VA USA; 3 UVA Comprehensive Cancer Center, 1300 Jefferson Park Avenue, West Complex Room 6009, PO Box 800716, Charlottesville, VA 22908 USA

**Keywords:** Inotuzumab ozogamicin, Acute lymphoblastic leukemia, Measurable residual disease, Blinatumomab, CAR t-cell, Veno-occlusive disease

## Abstract

**Purpose of Review:**

Acute lymphoblastic leukemia (ALL) is a rare hematologic malignancy with a bimodal distribution of incidence in both pediatric/young adult and elderly patient populations. Despite the high complete remission rate, there is a high rate of relapse necessitating a need for therapy options in the relapsed/refractory setting. Given this, treatment paradigms for ALL have shifted towards targeted therapies and away from high-intensity chemotherapy. The efficacy of inotuzumab ozogamicin (InO) in the relapsed/refractory setting for pediatric and adult populations has led to incorporation of this targeted therapy into frontline regimens. In this review, the role of InO in the frontline, measurable residual disease (MRD) positive and relapsed/refractory settings is highlighted.

**Recent Findings:**

InO is a directed antibody-drug conjugate that binds to CD22 on the surface of leukemic blasts. The cell internalizes InO, prompting enzymatic cleavage in the lysosome that releases calicheamicin, inducing double-strand DNA breaks and causing apoptosis. However, off-target effects can lead to severe adverse events such as hepatotoxicity, including veno-occlusive disease, and myelosuppression. Prior studies have supported its use in the relapsed or refractory treatment setting; however, newer studies incorporating InO in the frontline have shown promising results. Newer studies have also shown evidence of utilization of InO in specific sub-populations of B-cell ALL, including those with MRD-positive disease and Philadelphia-positive (Ph +) disease, and as bridging therapy with CAR T-cell therapy, and in the post-transplant maintenance setting.

**Summary:**

This review evaluated the effectiveness of InO in clinical practice, associated adverse events, future directions in specific patient populations. Despite recent advancements, patients with B-cell ALL tend to have poorer outcomes, especially in the adult population. Future research and larger scale prospective studies are indicated to evaluate the efficacy of InO in different lines of therapy.

## Introduction

Acute lymphoblastic leukemia (ALL) is a hematologic malignancy with approximately 6,000 new cases a year that occurs most commonly in the pediatric and young adult population [1, 2]. The cure rates for young adults and children approach 90%, but the prognosis for adult ALL is much poorer. Despite complete remission rates in the 60–90% range, the high rate of relapse in adult ALL portends an overall poor prognosis with a 5-year overall survival (OS) between 40–50% [2–5]. With the use of targeted therapies like inotuzumab ozogamicin (InO), blinatumomab and CAR-T cell therapy, there has been an improvement in outcomes of patients with relapsed/refractory (R/R) disease. The role of measurable residual disease (MRD) negative status after induction in ALL has been well established. The success of these agents in the R/R setting has led to the incorporation of targeted therapies into frontline regimens in efforts to improve MRD negative outcomes [2, 6–8]. This review focuses on the mechanism of action, current use, and future directions of InO, an antibody drug conjugate directed to CD22, which is expressed on most B-cell malignancies such as B-ALL. InO is currently approved by the US Food and Drug Administration’s (FDA) for R/R B-cell ALL in both the pediatric and adult populations [9, 10].

## Drug Information

### Mechanism of Action

InO is a CD22-directed antibody drug conjugate (ADC) with a cytotoxic payload. Calicheamicin is covalently bound to a humanized anti-CD22 monoclonal antibody that is released when InO is internalized by the cell. Calicheamicin is then released, thereby inducing double-strand DNA breaks that result in apoptosis [11, 12].

### Adverse Effects

#### Hepatotoxicity, Including Hepatic Veno-Occlusive Disease

In the INO-VATE ALL trial, hepatotoxicity occurred in 14% of patients (23/164) during or following treatment with InO, or after subsequent allogeneic hematopoietic stem cell transplant (HSCT). This included hyperbilirubinemia, increased aspartate aminotransferase (AST), increased alanine aminotransferase (ALT) and veno-occlusive disease (VOD). InO causes VOD through direct endothelial injury mediated by calicheamicin-induced DNA damage and apoptosis in liver sinusoidal endothelial cells (LSECs). This then leads to sinusoidal congestion and narrowing, hepatocyte necrosis, and fibrosis of terminal hepatic venules, which collectively contribute to hepatic VOD [13, 14]. Among all patients who were treated with InO but did not proceed to HSCT, VOD occurred in 3% of the patients during treatment or follow-up. In contrast, VOD occurred in 23% of patients who received InO and proceeded to HSCT. The median time from HSCT to onset of VOD was 15 days. The use of dual-alkylator conditioning regimens and a total bilirubin level greater than or equal to the upper limit of normal (ULN) immediately prior to HSCT are significantly associated with an increased risk of developing VOD after HSCT. Other risk factors for VOD include increased age, greater number of InO treatment cycles, ongoing or prior liver disease, and prior HSCT [11, 12].

Given these risks, patients should be monitored closely for signs and symptoms of hepatotoxicity, including VOD. These include hyperbilirubinemia, ascites and rapid weight gain. Patients that are proceeding to HSCT should be limited to two cycles of InO prior to HSCT. If patients have not achieved complete remission (CR) or complete remission with incomplete recovery (CRi) and measurable residual disease (MRD) negativity prior to HSCT after 2 cycles, a third cycle of InO may be considered [11]. To prevent hepatotoxicity, it is recommended to wait 5 to 6 weeks from the last dose of InO before proceeding to HSCT [1, 15]. Ursodiol prophylaxis may also be considered during InO treatment as well as after HSCT to prevent hepatotoxicity including VOD [3, 16, 17]. Defibrotide is approved for the treatment of VOD in patients with renal or pulmonary dysfunction after HSCT, but data on the effectiveness in patients with VOD after InO is limited [18]. If a patient develops VOD during InO treatment, then the drug should be permanently discontinued [1].

#### Myelosuppression

Myelosuppression is a common occurrence in patients receiving InO. In the INO-VATE ALL trial, thrombocytopenia occurred in 51% of patients, with grade 4 thrombocytopenia occurring in 28% of patients [12]. Prolonged thrombocytopenia beyond 6 weeks was common [10]. Mechanistically, the cytotoxic effects of the calicheamicin payload of InO causes direct myelosuppression leading to the significant cytopenias [19]. Additionally, preclinical models have demonstrated that antibody-calicheamicin conjugates can cause target-independent damage to liver sinusoidal endothelial cells, leading to platelet sequestration within hepatic sinusoids and contributing to both acute and chronic thrombocytopenia [20].Neutropenia occurred in 49% of patients, with grade 4 neutropenia occurring in 27% of patients [12]. Febrile neutropenia was reported in 26% of patients and infections occurred in 48% of patients. However, fatal infections occurred in only 5% of patients. Infection types reported were fungal, bacterial and viral [1, 2]. Hemorrhage also occurred in 33% of patients with grade 3 or 4 hemorrhage in 5% of patients. The most common type of hemorrhage was epistaxis [11]. Patients receiving InO should be monitored closely for myelosuppression, and complete blood counts should be monitored prior to each dose. Prophylactic antimicrobials should be considered, and treatment interruptions may be needed, particularly for absolute neutrophil counts less than 1,000/mm^3^ and/or platelets less than 50,000/mm [3, 11, 12].

#### Other Adverse Reactions

Other notable InO toxicities include increased risk of post-transplant non-relapse mortality, infusion reactions, QTc prolongation, nausea and headache [1, 2]. In the INO-VATE ALL trial, a higher post-HSCT non-relapsed mortality was observed in the InO group (39%) compared to the investigator’s choice of chemotherapy group (23%). The most common cause of post-HSCT non-relapse mortality was VOD and infections [1, 2]. Grade 2 infusion reactions were reported in just 2% of patients receiving InO, but it is recommended to pre-medicate with a corticosteroid, antipyretic, and antihistamine prior to each InO dose and monitor patients for at least 1 h after the infusion [1]. QTc prolongation, defined as an increase *≥* 60 msec from baseline using the Fridericia formula, occurred in 3% of patients. An electrocardiogram should be obtained prior to initiating InO, after starting other medications known to prolong QTc, and then periodically as clinically indicated [1].

### Role in Relapsed/Refractory B-ALL

Currently, InO has the FDA approval for the treatment of adults and pediatric patients with R/R B-ALL. This approval was based on the pivotal Phase III INO-VATE Study which evaluated outcomes between InO and standard-of-care chemotherapy (SoC) in patents with relapsed/refractory ALL. Patients were randomized to either receive InO (*n* = 164) vs. SoC (*n* = 162), and all patients were required to have > 5% bone marrow blasts and have received 1–2 previous induction chemotherapy regimens for ALL. The complete remission (CR) or complete remission with incomplete hematologic recovery (CRi) was higher with InO compared to SoC (73.8% vs. 30.9%, *p* < 0.0001). The median overall survival (mOS) was 7.7 months with InO compared to 6.2 months with SoC (HR 0.75, *p* = 0.0105). The best predictors of OS with InO were thought to include the MRD status, duration of first remission, baseline platelet count, achievement of CR/CRi, and follow-up HSCT. The progression free survival (PFS) was 5 months in patients who received InO compared to 1.7 months in patients who received SoC. Furthermore, more patients who received InO proceeded directly to HSCT after achieving CR/CRi before any follow-up induction therapy compared to patients who received SoC (39.6% vs. 10.5%, *p* < 0.0001) [10].

The role of InO is also being evaluated in the R/R setting for patients with Philadelphia-positive (Ph+) ALL. The Philadelphia chromosome (Ph) is the most common cytogenetic abnormality in ALL. Ph + ALL is associated with worst outcomes and higher rates of relapse compared to Ph-negative (Ph-) patients, despite the use of tyrosine kinase inhibitors (TKIs). InO has shown promise in Ph + ALL, although the data is mostly limited to pooled retrospective analyses of large trials that include both Ph + and Ph- patients. Stock et al. analyzed the outcomes in Ph + ALL patients in both the INO-VATE phase III trial as well as a phase I/II dose-finding study conducted by DeAngelo et al. which also investigated InO in patients with R/R ALL. The rates of CR/CRi were significantly higher in patients who received InO compared to SoC in the INO-VATE trial (73% vs. 56%). Additionally, the rate of MRD negativity was significantly higher in the InO group compared to SoC (81% vs. 33%). This led to twice as many patients proceeding HSCT compared to the SoC group (41% vs. 19%) [10, 21]. Similarly, the aforementioned phase I/II study also demonstrated significantly higher rates of CR/CRi in patients who received InO compared to SoC [22]. Jain et al. most recently conducted a phase I/II trial investigating the addition of bosutinib to InO in patients with R/R Ph + ALL and blast-phase chronic myeloid leukemia (CML). Of note, patients with a T315I mutation were excluded. Bosutinib was given at three daily dose levels (300 mg, 400 mg, 500 mg) while InO was dosed weekly during cycle 1, then monthly subsequently for a total of 6 cycles. Among the 18 patients, the investigators concluded that this combination was overall well tolerated with the maximum tolerated dose of 400 mg daily for bosutinib. The median duration of response was 7.7 months and OS was 13.5 months (with a median follow-up of 44 months). The CR/CRi rate was 83%. These findings support the addition of bosutinib to InO for this patient population [23]. 

### Role in Frontline B-ALL

Most recent smaller studies have focused on the use of InO in the frontline setting, focusing on patients who are not candidates for intensive chemotherapy and in intensive pediatric based regimens. These trials, summarized below, investigate the combination of InO with low-intensity chemotherapy, blinatumomab or as a single-agent combined with dexamethasone.

#### Inotuzumab Ozogamicin and Low-Intensity Chemotherapy

A phase II study from Kantarjian et al., evaluated the efficacy and safety of adding InO to low-intensity chemotherapy in elder patients with ALL. The trial included 52 patients over the age of 60 with newly diagnosed Ph- B-ALL. Patients received low-intensity chemotherapy mini-hyper CVD. Odd-numbered cycles included IV cyclophosphamide, oral or IV dexamethasone, and IV vincristine. No anthracycline was given. Even-number cycles included IV methotrexate (MTX) and IV cytarabine. Cycles were administered every 4 weeks for a total of 8 cycles, with InO being administered on day 3 of each of the 4 cycles. Notably, the dosing of InO varied, as a “run-in” phase was performed to determine the dose-limiting toxicities and maximize the tolerated dose of InO in combination with low-intensity chemotherapy. InO was dosed at 1.3-1.3.8mg/m^2^ for cycle 1 followed by 1.0-1.0.3mg/m^2^ for subsequent cycles. Maintenance therapy was given for three years through the dose-reduced POMP regimen (6-Mercaptopurine, vincristine, MTX, prednisone) [24].

The 2-year event-free survival (EFS) rate and OS rates were 59% and 66% respectively, with a median follow-up period of 29 months. No early death was reported within 4 weeks, and fewer deaths in patients with complete remission were observed compared to historical published data with hyper-CVAD in an elderly population (23% vs. 34% respectively) [25]. Every patient on the study had hepatic adverse effects of any grade, including at least grade 3 events of 33%. 8% developed VOD after a median of three cycles, and notably, all 4 cases were encountered in patients who received the higher dose of InO. This study demonstrates the safety and efficacy of combining low-intensity chemotherapy with InO in older patients with newly diagnosed Ph- ALL [8].

#### Adding Inotuzumab Ozogamicin to a Pediatric Inspired Chemotherapy Regimen

The SoC for adolescents/young adult (AYA) patients with newly-diagnosed ALL (age 18–39) is pediatric inspired chemotherapy regimens which have shown overall improved survival rates [26, 27]. A recent randomized phase III Alliance cooperative group trial studied the addition of InO to the pediatric inspired regimen CALGB 10,403 in the AYA population with newly-diagnosed B-ALL. The CALGB 10,403 regimen was slightly modified in that the extended induction course was omitted, the pegaspargase dose was capped to 3750 units, and rituximab was given to patients with CD20 expression greater than 20%. Additionally, patients who had MRD positivity were given blinatumomab. Patients who achieved CR/CRi or partial remission (PR) were either randomized to receive two cycles of InO (1.5mg/m^2^/cycle) or continue the CALGB 10,403 backbone. The trial did not meet its primary EFS endpoint; 3-year EFS was 69.0% in the InO arm and 66.7% for the control arm (HR 0.97; 0.58–1.63). It also did not meet its OS secondary endpoint. Additionally, the InO arm had significantly more toxicities reported compared to the control arm. Despite the negative results of this study, there may be a role still for InO in combination with pediatric inspired therapies if toxicities can be mitigated [28].

#### Inotuzumab Ozogamicin and Blinatumomab

A study from Senapati et al. evaluated the efficacy of a chemotherapy-minimized approach with InO and blinatumomab. Fourteen patients (age > 70 or > 60 but unfit) with Ph- B-ALL were evaluated in this study. During cycle 1, patients received IV dexamethasone from days 1–4, IV vincristine on day 4, fractionated InO on day 1 and day 8, and continuous blinatumomab from day 15 for 14 days. This was followed by 4 consolidation cycles with continuous blinatumomab for the first 28 days of the 42-day cycle with InO (0.6mg/m^2^) on days 1 and 8. Afterwards, patients received 4 cycles of maintenance single-agent blinatumomab. At median follow-up time of 15 months, the median PFS and OS were not reached. At 1 year, PFS and OS were 64% and 73% respectively. At the data cutoff, 5 patients died (2 after relapse and 3 in remission). One patient had a grade 3 ALT elevation, but no patients developed hepatic sinusoidal obstruction syndrome, and no patients developed a secondary myeloid neoplasm. The study concluded that a chemotherapy-minimal combination of InO and blinatumomab leads to promising response and survival outcomes in older patients with newly diagnosed B-ALL that appears to be relatively tolerable [29].

The Alliance study from Wieduwilt et al. was a phase II study to evaluate the effect of induction with InO followed by blinatumomab in older adults (age > 60) with newly diagnosis Ph- B-ALL with CD22+ (> 20% of blasts) without plans for HSCT. All patients were induced with InO (0.8 mg/m^2^ on days 1 and 8, and 0.5 mg/m^2^ on day 15) of a 21-day cycle. Cytoreduction was evaluated afterwards, defined as marrow blasts by > 50% or cellularity < 20%. Patients without cytoreduction to InO or if they did not have any events started blinatumomab consolidation, defined as 9 mcg/day for 7 days followed by 28 mcg/day for 21 days, again followed by a 14-day break and 28 mcg/day for 28 days. Patients who had CR/CRi received two subsequent maintenance cycles of blinatumomab while others received three more cycles. Thirty-three patients were evaluated in the study and the cumulative CR rate after InO induction versus after blinatumomab maintenance was 85% and 97% respectively. EFS was defined from therapy start to failure to achieve CR by the end of blinatumomab consolidation, progression, death, or relapse. With a median follow-up period of 22 months, the 1-year EFS and OS were 75% and 84% respectively. Twelve patients had events with 9 relapses, 2 deaths in remission, and 1 death from respiratory failure with sinusoidal occlusion of the liver. This study showed that InO induction followed by subsequent blinatumomab consolidation can be efficacious for newly diagnosed Ph- B-ALL with CD22 + [30].

#### Inotuzumab Ozogamicin Single-Agent Induction

Stelljes et al. conducted the INITIAL-1 phase II trial with 45 patients over the age of 55 with newly diagnosed CD22 + B-ALL to evaluate the impact of InO and dexamethasone induction followed by consolidation and maintenance therapy. The primary endpoint was EFS at 12 months of follow up after the initiation of induction therapy, with an event defined as persistence of > 5% bone marrow blasts after two cycles of InO, hematological relapse, or death [31].

Up to three cycles of InO induction was given if the peripheral blast count was < 10% after 5 days of prephase treatment with dexamethasone and low-dose cyclophosphamide. The first 21-day induction cycle included 0.8 mg/m2 of InO on day 1 and 0.5 mg/m^2^ on day 8 and day 15 in addition with 10 mg/m^2^ of dexamethasone on days 7–8 and 14–17. One intrathecal injection of MTX, cytarabine, and dexamethasone was given during the first cycle. During the second and third 28-day induction cycles, patients received 0.5 mg/m^2^ of InO on days 1, 8, 15, and one intrathecal application of MTX, cytarabine, and dexamethasone per cycle. Patients who achieved CR were offered up to five cycles of age-adapted consolidation [32]. Patients also received rituxumab for CD20 + disease (> 20% CD20-positive blasts at the time of diagnosis). After consolidation, patients received maintenance therapy with 60mg/m^2^/day of oral mercaptopurine and 20mg/m^2^/week of MTX up to a total duration of treatment of 2 years.

All patients were able to achieve CR/CRi, with 98% of patients completing three cycles of InO induction treatment. 53% and 71% of patients did not have evidence of MRD after the second and third inductions, respectively. With a median follow-up period of 2.7 years, the EFS at 1 and 3 years were 88% and 55%, while the OS at 1 and 3 years were 91% and 73% respectively. The most common adverse effects included grade III cytopenias and elevated liver enzymes. Overall, this study demonstrated the tolerability and efficacy of InO-based induction followed by age-adapted chemotherapy with high rates of remission and OS, providing data to support integration of InO into first-line regimens for elderly patients with B-ALL [31].

### Role of Inotuzumab Ozogamicin in MRD-positive Disease

The persistence of MRD after treatment for ALL is associated with elevated risk of relapse and increased mortality rate [33–35]. As such, studies looking at treatment modalities to eradicate MRD after the achievement of CR/CRi are underway. InO has demonstrated efficacy in patients who have achieved CR/CRi but with persistent or rising MRD. Jabbour et al. conducted a phase II study to evaluate the role of InO in eradicating MRD in patients with ALL who achieved CR but with persistently positive MRD [35]. These patients received a fractionated, lower-dose regimen consisting of6 cycles of InO (with each cycle lasting 21–28 days). Patients received 0.6mg/m^2^ on day 1 and 0.3 mg/m^2^ on day 8 of cycle 1.For cycles 2–6, patients received 0.3 mg/m^2^ on days 1 and 8.Patients with Ph+ disease also received a BCR::ABL1 TKI. The results were overall promising, with 69% of patients converting to MRD-negative status, with a large majority of these patients responding after 1 cycle of treatment. Of note, approximately half of the patients in this study had prior blinatumomab exposure, indicating that InO remains effective in patients who were previously exposed to MRD-directed therapies [35]. An earlier phase 2 study conducted by Marconi et al. in 2022 (Gimema ALL2418) also investigated the use of InO in both Ph- and Ph + ALL patients who have previously received at least one line of therapy with persistently positive MRD [36]. In contrast to the Jabbour et al. study, these patients received 1 cycle of InO only, with the option to receive an additional cycle of MRD negativity was not obtained with just one cycle. Overall, this study demonstrated a lower MRD eradication rate of 35%, which could be explained by several factors including the lower number of cycles received, as well as difference in MRD assays used between the two studies. Both studies showed that treatment with InO demonstrated activity (even in patients who previously received blinatumomab) with minimal adverse events. Indeed, InO is now included as an option on the NCCN guidelines for MRD positive patients. Confirmatory studies for mature efficacy and survival data are needed and will further elucidate how this promising treatment strategy can be integrated within the current treatment landscape for ALL. Studies evaluating a InO approach vs. blinatumomab approach for MRD positive patients are needed (Table 1).

**Table 1:** Summary of key clinical trials that support the use of InO in the treatment of ALL

Regimen (study)	Study design	Patient population	Key findings	Outcomes
Frontline				
InO+mini-hyperCVD (Kantarjian et al.)	Phase II, single-center study. Patient received HyperCVD followed by maintenance dose-reduced POMP. InO given on day 3 of the first 4 cycles.	Age ≥ 60. Newly diagnosed Ph- B-cell ALL.	2-year PFS: 59% (95% CI 43–72) 2-year OS: 66% (95% CI 50–78) Median follow-up period of 29 months	This study demonstrates the safety and efficacy of combining low-intensity chemotherapy with InO in older patients with newly diagnosed Ph- ALL.
InO+CALGB 10,403 (Alliance)	Phase III cooperative group trial. Patients were randomized 1:1, if they achieved CR/CRi or PR after CALGB 10,403 induction, to InO or continue CALGB 10,403 backbone	Age 18–39 years with newly diagnosed Ph- B-cell ALL.	Did not meet its primary EFS endpoint or OS secondary endpoint. InO arm had significantly more toxicities compared to the control arm.	InO in combination with pediatric inspired therapies is not recommended given significant toxicities.
InO+blinatumomab (Senapati et al.)	Phase II single-center study. Patients received induction with dexamethasone and vincristine, followed by blinatumomab. Consolidation cycles consisted of blinatumomab and InO	Age ≥ 70 (or 60–70 years unfit) with newly diagnosed Ph- B-cell ALL.	At median follow up time of 15 months, the median PFS and OS were not reached. 1-year PFS: 64% 1-year OS 73%	This chemotherapy-free approach has promising outcomes with minimal side effects in older patients.
InO+blinatumomab (Alliance)	Phase II cooperative group study. Patients received up to two cycles of InO followed by 4–5 cycles of blinatumomab	Age ≥ 60. Newly diagnosed Ph- B-cell ALL.	CR/CRi (InO after two cycles of InO vs. after two cycles of blinatumomab maintenance): 85% vs. 97% 1-year EFS: 75% (95% CI, 61–92) 1-year OS: 85% (95% CI 73–98)	that InO induction followed by subsequent blinatumomab consolidation can be efficacious for newly diagnosed Ph- B-ALL
InO-based induction (INITIAL-1)	Phase II, open-label, cooperative group trial. Patients received prephase treatment with dexamethasone, low-dose cyclophosphamide, followed by 3 cycles of InO and dexamethasone. Patients achieving CR received 5 cycles of age-adapted consolidation followed by maintenance with oral mercaptopurine and MTX.	Aged ≥ 56. Newly diagnosed Ph- B-cell ALL.	CR/CRi: 100% 71% of patients were MRD negative after 3 cycles of induction. 3-year EFS: 55% (95% CI, 40 to 71) 3-year OS: 73% (95% CI, 59 to 87)	The high remission rates and OS provide a rationale for the inclusion of InO into first-line regimens for older patients.
*Relapse/refractory*				
InO (INO-VATE)	Phase III, open-label. Randomized 1:1 to InO or investigator’s choice of SOC	Age ≥ 18 with relapsed/refractory Ph + or Ph- B-cell ALL having received 1–2 previous induction chemotherapy regimens.	CR/CRi (InO vs. SOC): 73.8% vs. 30.9% mOS (InO vs. SOC): 7.7 months vs. 6.2 months (HR 0.75, *p* = 0.0105)	Led to the approval of InO in the R/R setting
InO+bosutinib (Jain et al.)	Phase I/II, open-label, single-center study. Patients received bosutinib daily with dose escalation in a standard 3 + 3 design. InO was given on days 1, 8 and 15 of cycle 1, and on day 1 of cycles 2–6 (for a total of 6 cycles).	Age ≥ 18 with relapsed/refractory Ph + B-cell ALL. Excluded patients with T315I mutation and who had prior exposure to anti-CD22 directed therapy.	CR/CRi: 83% OS: 13.5 months (95% CI 7.5, not reached) Maximum tolerated dose of bosutinib: 400 mg daily.	Support the addition of bosutinib to InO for this patient population
*MRD-positive disease*				
InO (Jabbour et al.)	Phase II, open-label, single-center study. Patients received a fractionated, lower-dose regimen consisting of 6 cycles of InO.	Age ≥ 18 with Ph + and Ph- B-cell ALL who achieved first CR or beyond with either failure of MRD response or MRD recurrence.	2-year RFS: 54% (95% CI, 32–72) 2- year OS: 60% (95% CI, 36–77) MRD negativity: 69%	InO demonstrated favorable survival and rate of MRD negativity in patients with MRD-positive disease.
InO (Gimema ALL2418)	Phase II, open-label, multicenter study. Patients received one cycle of InO with the option to receive additional cycles if there was persistent MRD.	Age ≥ 18 with Ph + and Ph- B-cell ALL who achieved CR after at least one line of therapy.	MRD negativity: 35%	Conversion of MRD-positive to MRD-negative disease can be achieved with InO, likely with more than one cycle of InO.

*ALL* acute lymphoblastic leukemia, *CR* complete remission, *CRi* complete remission with incomplete hematologic recovery, *EFS* event free survival, *HyperCVD* cyclophosphamide, dexamethasone, vincristine, methotrexate, cytarabine, *InO* Inotuzumab Ozogamicin, *MRD* measurable residual disease, *PFS* progression free survival, *Ph* Philadelphia chromosome, *POMP* 6-mercaptopurine, vincristine sulfate, methotrexate, and prednisone, *OS* overall survival, *RFS* relapsed free survival, *SOC* standard of care

### Inotuzumab Ozogamicin in the Context of Other Available Therapies

#### CAR T-cell Therapy

In the R/R setting, autologous anti-CD19 chimeric antigen receptor (CAR) T-cell therapy has demonstrated a high rate of CR/CRi with improved OS [37]. InO has been used as bridging therapy or salvage therapy prior to CAR T-cell. A multicenter retrospective analysis by Aldoss et al. reported inferior outcomes in patients with R/R B-ALL who received InO prior to brexucabtagene autoleucel (brexu-cel). Of note, these associations were no longer significant in multivariate analyses, suggesting that the inferior outcomes in patients who received InO were unlikely to be due to exposure of the drug itself. Those who received InO had inferior PFS and OS. The timing of InO exposure – received as bridging therapy or before apheresis – did not affect CAR T-cell outcomes in this study [38, 39]. An earlier multicenter retrospective study from the UK demonstrated worse EFS in children and young adult patients age less than 26 who received bridging therapy with InO prior to tisagenlecleucel (RR 2.21; *P* = 0.03) [40].

These findings were challenged by a recent phase Ib/II study conducted by Park et al. which sought to characterize the impact of InO on lymphodepletion and pharmacokinetics of CAR T-cell therapy. Patients with R/R B-ALL received lymphodepletion with fludarabine and cyclophosphamide, followed by obecabtagene autoleucel CD19 autologous CAR T-cell. In this study, 14% (18/127) of patients received bridging therapy with InO, while the rest of patients received bridging therapy with another agent or no bridging therapy. InO led to a significant reduction in tumor burden at lymphodepletion (2% median blasts, vs. 52% in those who received bridging therapy without InO, 30% in those who did not receive bridging therapy). However, no differences in expansion and persistence of the CAR T-cell product were observed. Rates of grade 3 or more toxicities were low. This study suggests a role for InO in reducing blast burden prior to CAR T-cell therapy, although more studies with larger sample sizes are warranted [40, 41]. As we continue to learn more about the use of CAR T-cell therapy in this disease population, more studies are needed to elucidate the impact of InO on outcomes.

#### Post-Transplant Maintenance

The rates of relapse in patients with B-ALL who received HSCT range from 20–50% [42, 43]. As such, maintenance therapies after HSCT are being studied, including InO. A phase I multicentered study by Metheny et al. included patients with CD22 + B-ALL who were in CR after HSCT and had high risk of recurrence. The trial was designed in a typical “3 + 3” model to determine the maximum tolerated dose, safety and efficacy of InO. The maximum tolerated dose in this cohort was 0.6 mg/m^2^. Low-dose InO showed efficacy in the post-HSCT maintenance setting as demonstrated by a 1-year post-HSCT PFS of 89% and 1-year OS of 94%. Of the 14 patients studied, none had evidence of VOD or graft failure [44]. Future studies are needed to determine the optimal number of cycles that can be given after HSCT.

### InO in Clinical Practice

At our institution, InO in combination with mini-CVD is commonly used in the frontline setting for elderly or unfit patients with Ph- B-ALL. Patients who are young and fit receive pediatric-inspired chemotherapy regimens with either CALGB 10,403 (adolescent and young adult patients only) or HyperCVAD. Post-induction MRD is obtained to determine consolidation therapy. In patients who achieve a CR/CRi but with MRD-positive disease, blinatumomab is used to induce MRD negativity. InO is used for persistent MRD positivity despite blinatumomab. However, for patients who are candidates for HSCT, the use of InO in the frontline setting is limited to avoid the increase risk of VOD post-HSCT. In the relapsed/refractory setting, InO is used per its FDA approval.

## Conclusions

Despite recent advancements, B-cell ALL continues to have poor outcomes, especially in the adult population. This is especially true in patients with R/R disease, are elderly (age > 60) or are too unfit for standard multiagent chemotherapy regimens. This highlights the need for ongoing therapeutic advancements that provide safe and effective treatment options for this patient population. InO has been shown to be safe and effective in the R/R setting and is approved by the FDA for this indication. The studies that have led to this approval, as well as ongoing studies, have evaluated ways in which to mitigate severe adverse reactions from the medication, including VOD. Recent data has shown promising outcomes in the frontline setting when combined with low-intensity and pediatric regimen chemotherapy and blinatumomab, even in patients who do not receive HSCT. Further uses of this therapy are currently being explored, such as in combination with a TKI for patients with Ph + ALL, bridging therapy prior to CAR T-cell therapy, in MRD positive disease, and as post-transplant maintenance therapy.

## Key References

- Kantarjian H, Ravandi F, Short NJ, et al. Inotuzumab Ozogamicin in Combination with Low-Intensity Chemotherapy (mini-hyper-CVD) As Frontline Therapy for Older Patients with Philadelphia Chromosome-Negative Acute Lymphoblastic Leukemia: A Single-Arm, Phase II Study. *Lancet Oncol*. 2018;19(2):240–248. doi:10.1016/S1470-2045(18)30011-1.
  - This study evaluated the safety and efficacy of combining low-intensity chemotherapy with inotuzumab in older patients with newly diagnosed Ph- negative B-ALL. Although patients did have hepatic adverse effects, the data showed fewer deaths in patients with complete remission compared to those who historically received hyper-CVAD.
- Kantarjian HM, DeAngelo DJ, Stelljes M, et al. Inotuzumab ozogamicin versus standard of care in relapsed or refractory acute lymphoblastic leukemia: Final report and long-term survival follow-up from the randomized, phase 3 INO-VATE study. *Cancer*. 2019;125(14):2474–2487. doi:10.1002/cncr.32116.
  - This study showed that inotuzumab significantly improved the rates of complete remission, complete remission with incomplete recovery, and overall survival in adults with relapsed or refractory B-cell acute lymphoblast leukemia.
- Senapati J, Jabbour E, Short NJ, et al. Chemotherapy Free Regimen of Inotuzumab Ozogamicin and Blinatumomab in Frontline Therapy of Older Patients with Philadelphia Negative B-Cell Acute Lymphoblastic Leukemia. *Blood*. 2024;144(Supplement 1):1442. doi:10.1182/blood-2024-208742.
  - This study evaluated the efficacy of a chemotherapy-minimized approach with Inotuzumab and blinatumomab on elderly patients with Ph- B-cell ALL. It demonstrated promising outcomes with PFS and OS of 64% and 73% respectively at 1 year with relatively tolerable outcomes.

## Data Availability

No datasets were generated or analysed during the current study.
